# Tunable structural rearrangement in Cu cluster assemblies through linker and solvent alterations†

**DOI:** 10.1039/d4sc07730j

**Published:** 2025-01-22

**Authors:** Saikat Das, Jin Sakai, Riki Nakatani, Ayumu Kondo, Rina Tomioka, Subhabrata Das, Shuntaro Takahashi, Tokuhisa Kawawaki, Sourav Biswas, Yuichi Negishi

**Affiliations:** a Research Institute for Science & Technology, Tokyo University of Science Tokyo 162-8601 Japan sourav.biswas210@gmail.com; b Department of Applied Chemistry, Faculty of Science, Tokyo University of Science Kagurazaka, Shinjuku-ku Tokyo 162-8601 Japan; c Chemical Materials Development Department, Tanaka Precious Metal Technologies Co., Ltd, Tsukuba Technical Center 22 Wadai Tsukuba Ibaraki 300-4247 Japan; d Institute of Multidisciplinary Research for Advanced Materials, Tohoku University Aoba-ku Sendai 980-8577 Japan yuichi.negishi.a8@tohoku.ac.jp

## Abstract

The scarcity of approaches to assembling copper nanoclusters (Cu NCs) has restricted advancements in Cu NCs research, largely due to stability challenges of the individual NCs. By utilizing the structural adaptability of Cu NCs, we systematically investigate how variations in organic linkers and solvents affect the cluster node size, shape, and their assembling dimensionality. Here, we introduce a facile, one-pot synthesis method for obtaining a range of crystalline Cu cluster-assembled materials (CAMs) through a liquid–liquid interfacial crystallization technique. Our approach demonstrates that the electronic environment of linker molecules plays a crucial role in constructing the geometry of cluster nodes and the overall dimensionality of the framework. Solvent effects further influence the electronic environment of linkers, leading to tunable rearrangements in cluster node size and geometry. Additionally, coordination sites of the linker molecules and architectural properties significantly affect the overall dimensionality of the frameworks. Furthermore, correlations between solid-state photophysical properties and structural architecture expand the scope of this study, introducing the potential for tunable optical properties. We anticipate that this work will not only open avenues for designing novel Cu CAMs but also guide future research toward Cu-based materials with customizable optical features.

## Abstract 2

This study introduces a one-pot synthesis of crystalline Cu cluster-assembled materials using liquid–liquid interfacial crystallization, featuring Cu_6_, Cu_7_ and Cu_10_ cluster nodes connected by six organic linkers of varying dimensionalities.

## Graphical abstract

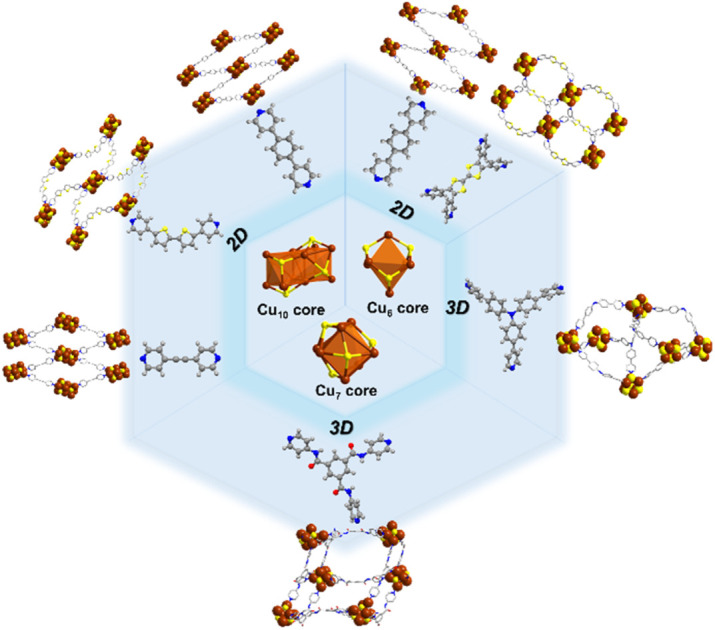

## Introduction

The synthesis of copper (Cu) nanoclusters (NCs) has emerged relatively recently compared to their gold (Au) and silver (Ag) counterparts, primarily due to the inherent challenges in stabilizing the NCs during synthesis, which are prone to oxidation and decomposition, especially at ambient conditions.^1–5^ However, recent advances in synthetic methodologies have made it possible to stabilize Cu NCs.^6,7^ Early efforts focused on Cu-hydride NCs, which are stable at low temperatures, but the development of thiolate- and alkyne-capped Cu NCs has since enabled the stabilization of these clusters under ambient conditions.^7–13^ After stabilization, Cu NCs exhibit remarkable properties arising from quantum confinement and their discrete electronic structures. These distinguish them from larger Cu nanoparticles, as the electronic and optical behaviors shift dramatically at the nanoscale, unlocking a wide array of potential applications.^14–22^ A key advantage of Cu NCs lies in the variable oxidation state of Cu and high surface-to-volume ratio enhances their versatility for catalytic applications.^23^ Additionally, the natural abundance and lower cost, compared to precious metals like Au and Ag, make Cu NCs highly attractive for practical and large-scale catalytic processes.

However, the lower reduction potential of Cu compared to noble metals like Au or Ag introduces substantial challenges even after the synthesis of Cu NCs.^23^ Unlike their more stable counterparts, Cu NCs are particularly prone to oxidation and aggregation, especially in catalytic environments where they encounter fluctuating reaction conditions.^6,24,25^ This increased susceptibility often leads to degradation of the Cu NCs, which manifests as reduced stability, reactivity, and catalytic efficiency over time. Recent efforts focus on tuning the surface chemistry to improve both functionality and stability.^26–29^ However, modifying surface ligands while maintaining core integrity remains a challenge, as the balance between oxidation protection and catalytic activity is delicate.

Thus, researchers are exploring alternative approaches, such as searching for robust ligands and designing stable frameworks, to improve Cu NC stability. A key influence is the successful assembly of Ag NCs using linker molecules, which has been highly effective in enhancing stability and expanding functionality.^30,31^ Applying this strategy to Cu NCs could open new possibilities for stabilizing and tuning their properties. The spatial constraints of peripheral ligands significantly impact cluster architecture, altering nuclearity and enabling the shift from discrete clusters to polymeric assemblies. However, comprehensive research on controlling the dimensionality of Cu NC assemblies through linker molecules and solvent conditions remains limited.^32–34^

In this context, we introduce a straightforward one pot-synthetic method to prepare seven Cu cluster assembled materials (CAMs) with varying dimensionalities through a liquid–liquid interface crystallization technique. Our findings demonstrate a clear relationship between the choice of linker molecules and the structural rearrangement of the cluster node, as well as the overall framework dimensionality. By utilizing different N-coordinating linkers, this approach provides a novel and versatile strategy to expand research in this area. Additionally, we uncover the significant influence of the solvent medium on the structural arrangement of the cluster node during synthesis which corelates with their solid-state photophysical properties. We believe that the ability to control and manipulate these factors could lead to major advancements in the development of Cu-based materials with tailored properties for specific applications.

## Experimental

### Synthesis of [CuS^*t*^Bu]_*n*_ complex

The synthesis of the [CuS^*t*^Bu]_*n*_ complex was initiated by treating a solution of copper(ii) nitrate trihydrate (3.0 mmol) in 10.0 mL of acetonitrile with 10.0 mL of triethylamine, resulting in the formation of a blue precipitate. With continuous stirring, *tert*-butyl mercaptan (9.0 mmol) was then added, dissolving the precipitate and producing a yellow solution. The solvent was then fully evaporated, and the resulting solid was washed with methanol and dried to yield a yellow precipitate as the final product, with an approximate yield of 95%.

### Synthesis of [[Cu_6_(S^*t*^Bu)_4_(CF_3_COO)_2_(dpb)_4_][Cu_6_(S^*t*^Bu)_4_(CF_3_COO)(dpb)_5_]]·CF_3_COO (Cu_6_-dpb) (dpb: 1,4-di(4-pyridyl)benzene) CAM

**Fig. 1:**
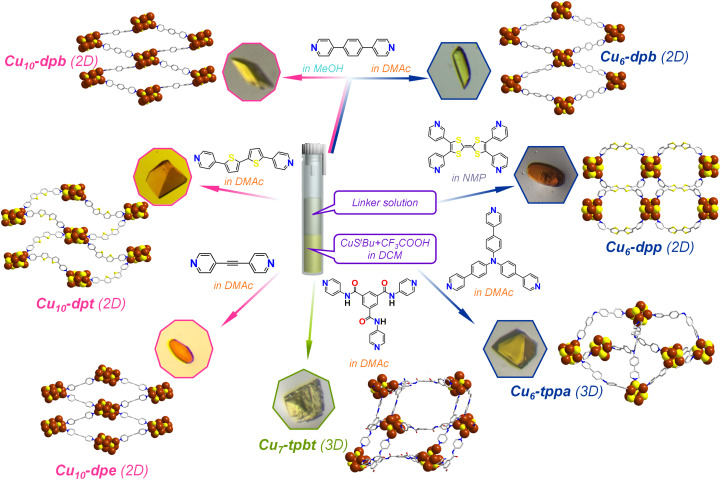
Schematic illustration depicting the one-pot synthesis of seven Cu CAMs composed of Cu_6_, Cu_7_ and Cu_10_ cluster nodes bridged by six different organic linkers. The effect of the solvent molecules is depicted by the specific color code. The optical microscope images represent the corresponding Cu CAM crystals. Hydrogen and all other non-essential atoms as well as the associated solvent molecules are removed for the clarity. Color legend: Cu, brown; S, yellow; O, red; N, blue; C, grey stick.

In a typical experimental procedure, 0.065 mmol of the [CuS^*t*^Bu]_*n*_ complex was first dispersed in 1 mL of dichloromethane (DCM) to create a uniform suspension. This suspension was then treated with 0.09 mmol of CF_3_COOH while stirring continuously at room temperature, until the solution became transparent. Afterward, 0.065 mmol of a freshly prepared dpb linker, dissolved in 1 mL of *N*,*N*-dimethylacetamide (DMAc), was gradually added to the reaction mixture dropwise. The entire solution was allowed to stand at room temperature, undisturbed, for a period of two days to enable crystallization. At the end of this period, rod-like yellow crystals (yield: 40.6% on the basis of Cu) formed and were observed at the interface between the two immiscible liquids. The crystal morphology and chemical composition were confirmed through optical microscopy and SEM imaging combined with EDS analysis (Fig. 1 and S1†).

### Synthesis of [Cu_10_(S^*t*^Bu)_6_(CF_3_COO)_4_(dpb)_4_] (Cu_10_-dpb) CAM

The typical reaction process followed a procedure similar to the one described previously, with the primary difference being the choice of solvent medium. In this modified approach, the initial complexation reaction was carried out in DCM as before; however, the linker solution was prepared in methanol (MeOH) instead of DMAc as previously used, and then added to the mixture. This seemingly small modifications in the solvent media significantly altered the resulting morphology of the crystals. The newly obtained rectangular parallelepiped-shaped yellow crystals (yield: 42.2% on the basis of Cu) were collected and set aside for further characterization to investigate the impact of the solvent changes on their structural and physical properties. The crystal morphology and chemical composition were confirmed through optical microscopy and SEM imaging combined with EDS analysis (Fig. 1 and S2†).

### Synthesis of [Cu_10_(S^*t*^Bu)_6_(CF_3_COO)_4_(dpe)_4_]·CH_2_Cl_2_ (Cu_10_-dpe) (dpe: 1,2-di(pyridin-4-yl)ethyne) CAM

The standard reaction process for synthesizing the (Cu_6_-dpb) complex was followed in this experiment, with the sole modification being the substitution of the dpb linker with dpe. This change was made to investigate how the chain length of the linker might influence the structure and crystallization behavior of the resulting complex. Following the reaction, the mixture was left undisturbed for two days to allow crystallization to occur. After this period, rice grain-shaped orange crystals (yield: 23.6% on the basis of Cu) were observed forming at the interface between the two immiscible liquid phases. The crystal morphology and chemical composition were confirmed through optical microscopy and SEM imaging combined with EDS analysis (Fig. 1 and S3†).

### Synthesis of [Cu_10_(S^*t*^Bu)_6_(CF_3_COO)_4_(dpt)_4_] (Cu_10_-dpt) (dpt: 5,5′-di(pyridin-4-yl)-2,2′-bithiophene) CAM

The standard reaction process for synthesizing the (Cu_6_-dpb) CAM was followed in this experiment, with the sole modification being the substitution of the dpb linker with dpt. Finally, we got truncated tetrahedron-shaped orange crystals (yield: 44.9% on the basis of Cu) were observed forming at the interface between the two immiscible liquid phases. The crystal morphology and chemical composition were confirmed through optical microscopy and SEM imaging combined with EDS analysis (Fig. 1 and S4†).

### Synthesis of [Cu_6_(S^*t*^Bu)_4_(tppa)_6_]·(CF_3_COO)_2_ (Cu_6_-tppa) (tppa: tris(4-(pyridin-4-yl)phenyl)amine) CAM

The standard reaction process for synthesizing the (Cu_6_-dpb) complex was followed in this experiment, with the sole modification being the substitution of the dpb linker with tppa which yielded truncated tetrahedron-shaped yellow crystals (yield: 25.1% on the basis of Cu). The crystal morphology and chemical composition were confirmed through optical microscopy and SEM imaging combined with EDS analysis (Fig. 1 and S5†).

### Synthesis of [Cu_7_(S^*t*^Bu)_4_(CF_3_COO)_2_(tpbt)_6_]·CF_3_COO (Cu_7_-tpbt) (tpbt: *N*1,*N*3,*N*5-tri(pyridin-4-yl)benzene-1,3,5-tricarboxamide) CAM

The standard reaction process for synthesizing the (Cu_6_-dpb) complex was followed in this experiment, with the sole modification being the substitution of the dpb linker with tpbt which yielded block-shaped yellow crystals (yield: 22.8% on the basis of Cu). The crystal morphology and chemical composition were confirmed through optical microscopy and SEM imaging combined with EDS analysis (Fig. 1 and S6†).

### Synthesis of [Cu_6_(S^*t*^Bu)_4_(CF_3_COO)_2_(dpp)_4_] (Cu_6_-dpp) (dpp: 3,3′-[2-(4,5-di-3-pyridinyl-1,3-dithiol-2-ylidene)-1,3-dithiole-4,5-diyl]bis[pyridine]) CAM

In this experiment, the standard synthesis process for the (Cu_6_-dpb) complex was followed. However, due to solubility issues with the dpp linker, it was dissolved in *N*-methyl-2-pyrrolidone (NMP) and gradually added to the reaction mixture. After two days, rice grain-shaped orange crystals were obtained (yield: 31.3% on the basis of Cu). The crystal morphology and chemical composition were confirmed through optical microscopy and SEM imaging combined with EDS analysis (Fig. 1 and S7†).

## Results and discussion

A one-pot synthetic process was adopted here to synthesize various Cu CAMs where the crystals were obtained through liquid–liquid interfacial crystallization process (Fig. 1). In each Cu CAM synthesis, the initial step involved a complexation reaction in which the [CuS^*t*^Bu]_*n*_ complex was treated with CF_3_COOH in DCM medium. Following the complexation, a solution containing different linker molecules was added to the reaction mixture, and the system was left to undergo crystallization. To induce more variation, the solvent used to dissolve the linkers was altered, creating a separate phase from the metal complex. The resulting crystals were characterized using single-crystal X-ray diffraction (SCXRD) to analyze their structural architecture.

**Fig. 2:**
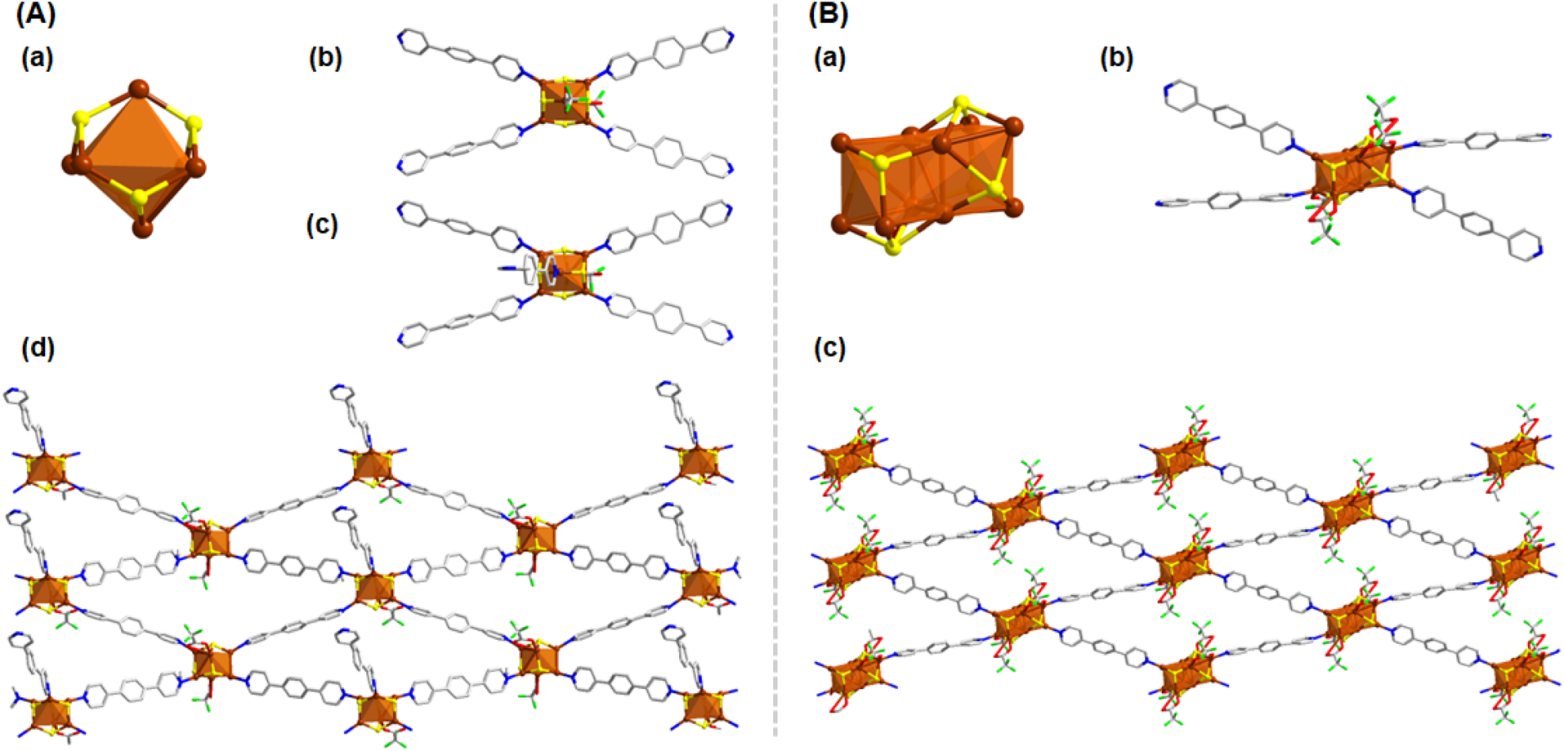
(A) Structural architecture of Cu_6_-dpb (a) Cu_6_ cluster node geometry and the attachment of four thiolate groups, (b) and (c) attachment of linker molecules and CF_3_COO^−^ units, (d) 2D architecture of the Cu_6_-dpb CAM. (B) Structural architecture of Cu_10_-dpb (a) Cu_10_ cluster node geometry and the attachment of four thiolate groups, (b) attachment of linker molecules and CF_3_COO^−^ units, (c) 2D architecture of the Cu_10_-dpb CAM. Hydrogen atoms and the associated solvent molecules are removed for the clarity. Color legend: Cu, brown; S, yellow; O, red; F, green; N, blue; C, grey stick.

According to this process when the simple bidentate dpb linker was introduced in a DMAc medium, Cu_6_-dpb crystals were obtained. The SCXRD analysis confirmed that the crystals formed in an orthorhombic system with the space group of *Pnma* (62). The detailed analysis reveals that the repeating cluster node consists of six Cu(i) atoms, each coordinated by four S^*t*^Bu^−^ ligands. These six Cu(i) atoms are arranged in an octahedral geometry, but with an average interatomic distance of 3.3488 ± 0.114 Å, which is too large to support significant cuprophilic interactions.^35^ Instead, the Cu(i) atoms are held together by thiolate bridges, where each thiolate ligand adopts a μ_3_ bridging mode, coordinating three Cu atoms simultaneously (Fig. 2A(a)). The average Cu–S bond distance in this arrangement is 2.2315 ± 0.00567 Å. The attainment of this specific structural geometry is not random; rather, literature repeatedly highlights the stability of the Cu_6_(SR)_6_ cluster node conformation, first reported in the Cu-thiolate nanocluster structure by Gao *et al.*^36^ Here, however, we observed two distinct attachment processes for an additional auxiliary ligand (CF_3_COO^−^) to the cluster node, linked to the binding of linker molecules. More detail analysis reveals that in one cluster node, only a single CF_3_COO^−^ unit is attached, along with five associated linker molecules. Conversely, in the adjacent cluster node, two CF_3_COO^−^ units are attached in opposite directions, accompanied by four linker molecules (Fig. 2A(b)). These two CF_3_COO^−^ units attach *via* a μ_1_ bridging mode. The linker molecules are attached through Cu–N bond where the four linker molecules are linked through mutual diagonal position whereas the additional linker molecule positioned opposite to the CF_3_COO^−^ unit (Fig. 2A(c)). The presence of these bidentate linker molecules causes the cluster nodes to act as repeating units in the overall structure. However, the diagonally attached four linker molecules are connected to the adjacent cluster nodes, the additional linker molecule, positioned opposite to the CF_3_COO^−^ unit, does not connect to any other cluster node but facilitates structural growth through interlayer stacking. Due to the linear nature of the linker molecules and their coordination to the cluster nodes, the extended structure forms a rhombohedral framework in a two-dimensional (2D) plane (Fig. 2A(d)). Each cluster node is separated by ∼15.315 Å along the axis, while the linker molecules themselves have a length of 11.378 Å. These 2D layers are stacked in an AB stacking fashion through non-covalent interactions, with a separation of approximately 9.726 Å between each layer.

A notable redistribution in the arrangement of Cu(i) atoms is observed when the solvent medium in the reaction is altered. In this case, the synthesis of the Cu_10_-dpb CAM proceeds *via* the dissolution of the linker molecules in a polar protic solvent, MeOH, as opposed to the previous use of a polar aprotic solvent (DMAc). This change creates an interface between the polar protic and aprotic solutions, initiating the redistribution of the Cu(i) atoms at the reaction environment. As a result, the reaction yields a structure consisting of ten Cu(i) atoms stabilized by six S^*t*^Bu^−^ ligands (Fig. 2B(a)). The rearrangement of Cu atoms also alters the number of attached CF_3_COO^−^ ligands and dpb linker molecules than the previous (Fig. 2B(b)). This observation underscores the crucial impact of the solvent environment on the distribution and stabilization of metal atoms during the synthesis process and its effect on the overall cluster node structure. The detailed structural architecture reveals that the larger interatomic distances (3.2829 ± 0.162 Å) limit the ability of the Cu(i) atoms to adopt a specific geometric arrangement. Despite this, strong cuprophilic interactions are observed, particularly in the middle Cu_4_ layer, where the Cu–Cu distance is measured at 2.6867 Å. This additional Cu_4_ layer is sandwiched between two Cu_3_ layers which are previously obtained in the Cu_6_-dpb, creating a notable structural feature. The detailed bond lengths and corresponding angles are compared with the Cu_6_ cluster nodes and the data reported in the literature (Table S8†). Among the six thiolate ligands, four engage in a μ_4_ bridging mode, connecting Cu(i) atoms between the middle and one of the Cu_3_ layer. The remaining two thiolates utilize a μ_3_ bridging mode, connecting Cu(i) atoms across the middle and one of the Cu_3_ layer. The average Cu–S bond distance is 2.2845 ± 0.0408 Å. The four CF_3_COO^−^ ligands are divided into two groups: two of them bridge the Cu(i) atoms *via* a μ_2_ bridging mode, while the other two adopt a μ_1_ bridging mode. The attachment of four dpb linkers results in the formation of a 2D framework structure, where the linkers are connected diagonally to the two Cu_3_ layers, similar to the previous arrangement (Fig. 2B(c)). Notably, the use of a polar protic solvent enables the incorporation of an additional four Cu(i) atoms, forming a middle layer. This middle layer is stabilized by two extra S^*t*^Bu^−^ ligands and two CF_3_COO^−^ ligands, providing further protection to the structure and balancing the overall contributory charge of the additional Cu(i). Anyway, again the arrangement forms a rhombohedral 2D framework where each cluster node is separated by ∼15.315 Å along the axis. Each 2D layers were further stacked in AB pattern through non-covalent interaction by maintain a separating distance of ∼7.291 Å. The absence of additional linker molecules in this structure, compared to previous results, leads to a shorter interlayer distance than observed in the earlier structure.

**Fig. 3:**
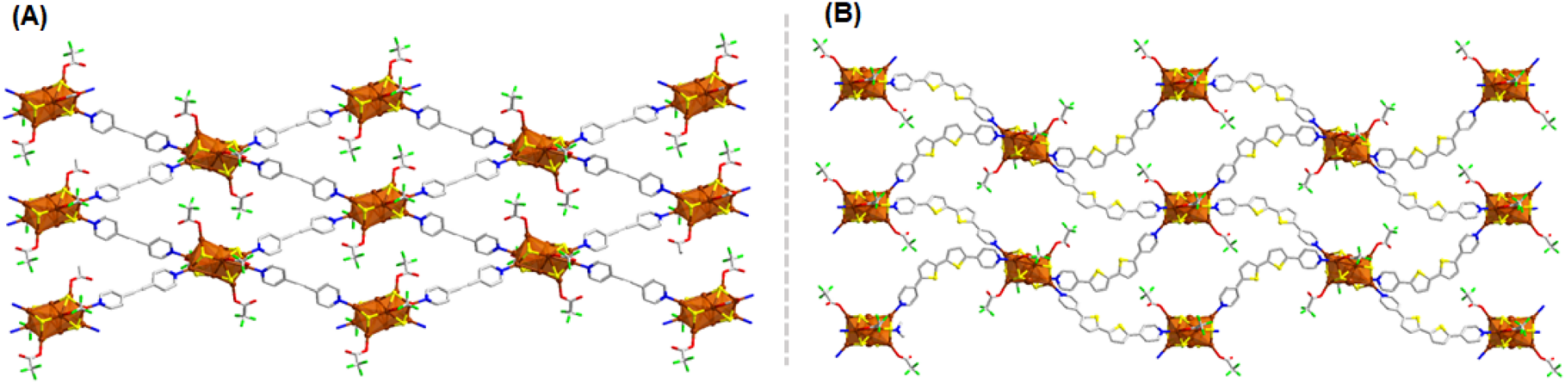
2D structural architecture of (A) Cu_10_-dpe (B) Cu_10_-dpt which are consisting similar Cu_10_ cluster node but different linker molecules. Hydrogen atoms and the associated solvent molecules are removed for the clarity. Color legend: Cu, brown; S, yellow; O, red; F, green; N, blue; C, grey stick.

Next, the impact of linker size is examined by using a shorter bidentate linear linker molecule compared to the previously employed one. Specifically, a dpe linker is utilized in place of the longer dpb linker. This substitution leads to the formation of Cu_10_-dpe CAM. The synthesis of Cu_10_-dpe CAM is carried out under reaction conditions identical to those used for the preparation of Cu_6_-dpb CAM, allowing for a direct comparison of the structural and functional effects of the different linker sizes. SCXRD analysis reveals that this Cu_10_-dpe CAM crystallizes in a monoclinic crystal system with a space group of *P*2_1_/*c* (14). The core structure of the cluster consists of ten Cu(i) atoms, which are stabilized by six S^*t*^Bu^−^ ligands and four CF_3_COO^−^ ligands, along with four dpe linker molecules resemblance to the structural architecture of Cu_10_-dpb CAM. So, a similar redistribution of Cu(i) atoms occurs with the incorporation of an additional Cu_4_ sandwiched layer, despite using the same solvent and surface-protecting ligands as before, indicating that the change in linker molecule drives this structural variation. Anyway, the cluster forms an extended 2D square framework (Fig. 3A), with individual cluster nodes spaced 13.6553 Å apart within the plane. The dpe linker itself measures 9.641 Å in length, suggesting that the shorter dpe linker favors the formation of a larger cluster node compared to the longer linker molecules. However, we noted a specific difference in this CAM compared to the Cu_10_-dpb CAM regarding the connection of the cluster node. In this case, we observed an alternate orientation of the adjacent cluster nodes that was not present before.

However, when a relatively larger, non-linear linker molecule, dpt, was introduced into the synthesis process using a similar reaction pathway, we observed the formation of a Cu_10_ cluster node structure once again. Due to the non-linear geometry of the linker, the resulting framework exhibited a distinctive wavy, two-dimensional network, in contrast to the more planar structures typically formed with shorter, linear linkers (Fig. 3B). Thus, both the shorter (dpe) and longer (dpt) linkers, when subjected to the same reaction protocol, led to the formation of a Cu_10_ cluster node. This observation suggests that the length of the linker molecule is not the primary determining factor for cluster node formation.^30,32^ Instead, the lone-pair donating capabilities of the pyridine moieties within the linker appear to play a more significant role in this process. To further explore this, we measured the zeta potential values of the linkers and observed that both the dpe (−13.9 mV) and dpt (−1.92 mV) linkers exhibited negative zeta potential values. In contrast, the dpb linker showed a positive zeta potential of 2.06 mV under neutral pH conditions (Fig. S8†). These differences in zeta potential suggest variations in the electronic environments of the linker surfaces, which could influence the overall behavior of the system. We assume that the negative zeta potential values of the dpe and dpt linkers arise from the stronger lone-pair donating capabilities of their pyridine groups. These electron-donating pyridine moieties may facilitate the aggregation of Cu(i) atoms into larger clusters, which ultimately results in variations in cluster size and structure. On the other hand, the positive zeta potential of the dpb linker likely reflects weaker electron donation, leading to smaller Cu(i) clusters. This nuanced interaction between the linker properties, particularly their electron-donating abilities, emphasizes a critical factor in the design of Cu(i) cluster-based frameworks. To further elucidate the rearrangement of the Cu_10_ cluster node in the Cu_10_-dpb CAM when altering the solvent environment compared to the Cu_6_-dpb CAM, we propose that the lone-pair donation ability of the pyridine moieties on the linker molecules is enhanced in MeOH as opposed to DMAc. This increased donation capacity in the MeOH medium likely facilitates the rearrangement process of the cluster node again, promoting a more stable and favorable structural configuration of the cluster node geometry.

**Fig. 4:**
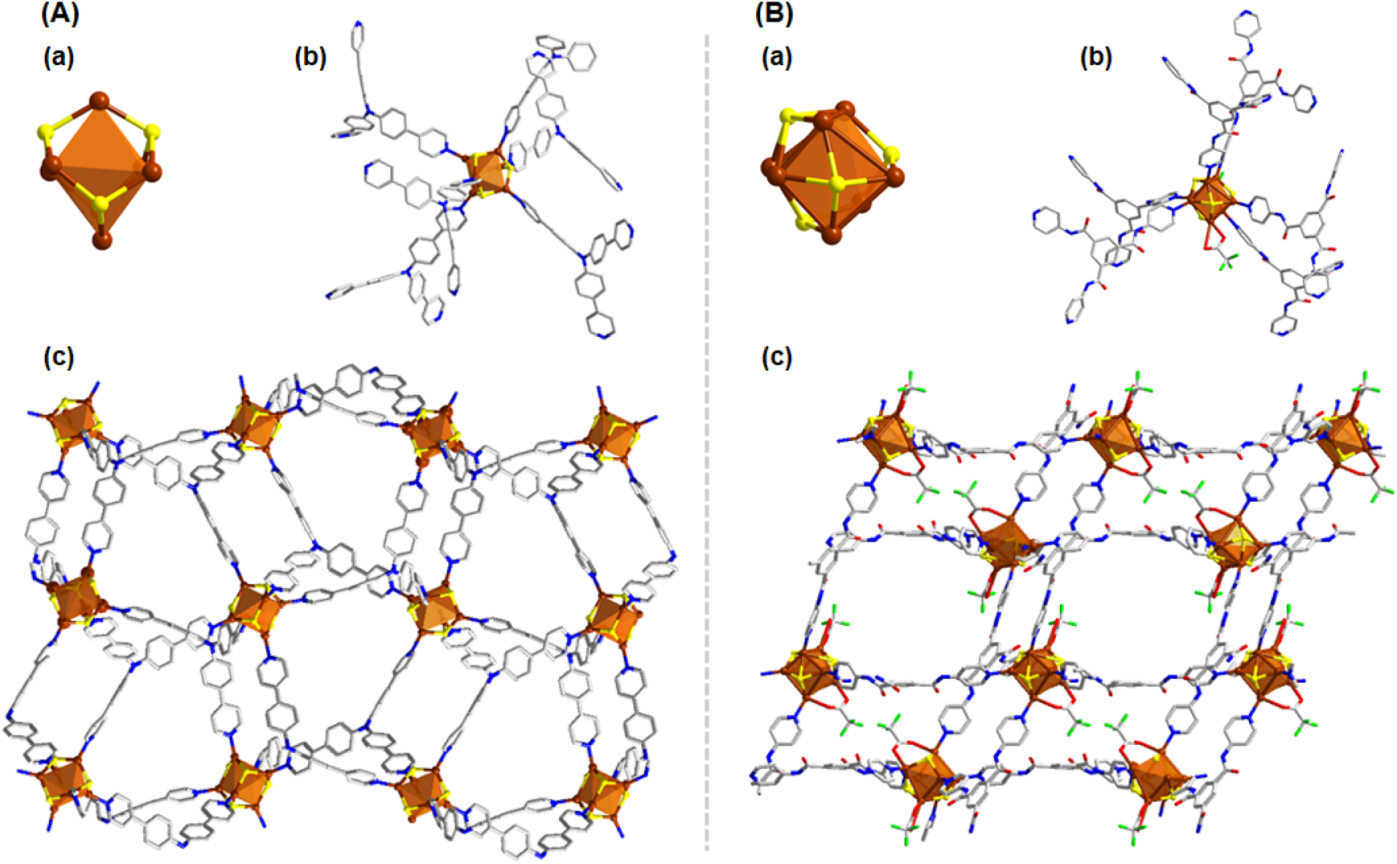
(A) 3D structural architecture of Cu_6_-tppa (a) Cu_6_ cluster node geometry and the attachment of four thiolate groups, (b) attachment of linker molecules, (c) 2D architecture of the Cu_6_-tppa CAM. (B) Structural architecture of Cu_7_-tpbt (a) Cu_7_ cluster node geometry and the attachment of four thiolate groups, (b) attachment of linker molecules, (c) 2D architecture of the Cu_7_-tpbt CAM. Hydrogen atoms and the associated solvent molecules are removed for the clarity. Color legend: Cu, brown; S, yellow; O, red; F, green; N, blue; C, grey stick.

By substituting the bidentate linker with the tridentate tppa, we initiated the formation of a three-dimensional (3D) structural framework, which had not been achievable with the previously used bidentate linkers. The reaction proceeded in a similar manner to our previous syntheses, yielding a Cu_6_ cluster node analogous to the previously characterized Cu_6_-dpb CAM. The slightly positive zeta potential value of the linker triggers a similar arrangement pathway of the cluster node, consistent with our earlier observations (Fig. 4A(a) and S9†); however, the attachment of six tppa linker molecules significantly altered its connectivity to the auxiliary ligands (Fig. 4A(b)). Instead of being attached to the cluster node, the CF_3_COO^−^ units were present as counter anions. Due to the tridentate nature of the linker molecule, each Cu_6_ cluster node is now connected to twelve neighboring cluster nodes from different planes, forming a robust 3D network (Fig. 4A(c)). The distance between adjacent cluster nodes was measured to be 17.402 Å, both along and across the axes.

In a subsequent modification, we replaced the tppa linker with another tridentate ligand, tpbt, to investigate its effect on the synthesis of the Cu CAM. This adjustment triggered a rearrangement of the Cu(i) atoms, resulting in the formation of a Cu_7_ cluster node (Fig. 4B(a)). This cluster node rearrangement is also influenced by the electronic environment of the linker molecules, which promotes the formation of larger cluster nodes with the negatively charged linker configuration (Fig. S9†). However, the negative zeta potential value may not be sufficient here to form the Cu_10_ cluster node, possibly due to the coordination site specificity of the linker molecules in the system. This new node consisted of four S^*t*^Bu^−^ ligands, two CF_3_COO^−^ ligands, and six tpbt linker molecules (Fig. 4B(b)). The additional Cu(i) atom incorporation to the cluster node opens up the possibilities to coordinating with the auxiliary ligands and thus CF_3_COO^−^ ligands were attached to the node. Similar to the previous framework, this Cu_7_ cluster node also forms a 3D network. However, in this case, each node connects to six neighboring nodes, resulting in a distinct structural arrangement (Fig. 4B(c)). Here the inter-node distance was found to be 19.097 Å, reflecting the expanded framework geometry.

**Fig. 5:**
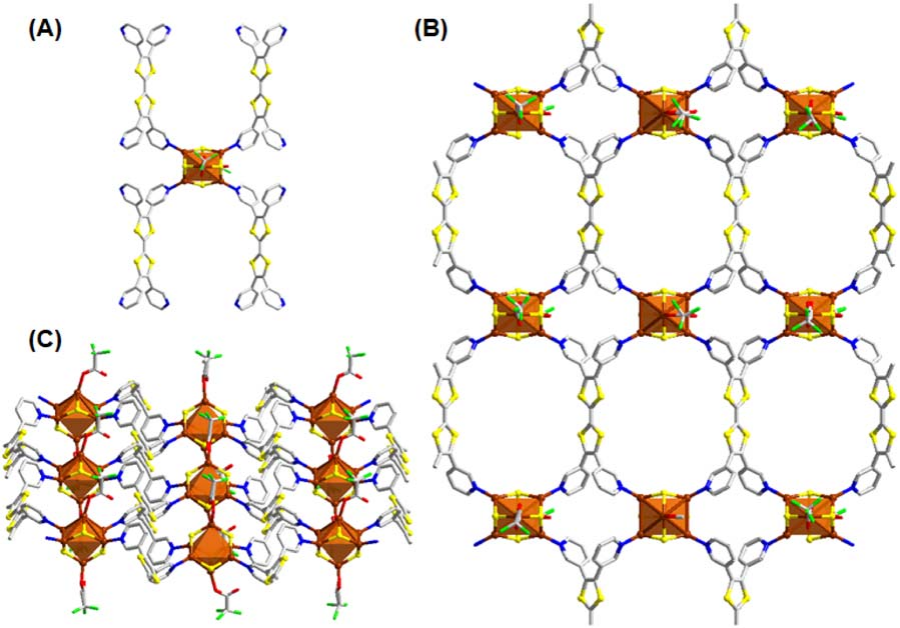
Structural architecture of Cu_6_-dpp (A) Cu_6_ cluster node geometry and the attachment of four thiolate groups, CF_3_COO^−^ units and the attachment of linker molecules, (B) 2D architecture of the Cu_6_-dpp CAM (C) ladder-like architecture. Hydrogen atoms and the associated solvent molecules are removed for the clarity. Color legend: Cu, brown; S, yellow; O, red; F, green; N, blue; C, grey stick.

After replacing the tridentate linker with the tetradentate dpp, we began forming a new 2D framework structure, Cu_6_-dpp. In this case, we dissolved the linker molecule in NMP instead of DMAc to resolve solubility issues; however, this change did not impact the size or shape of the cluster nodes, indicating the similar electronic environment of the linker molecule. We identified a positive zeta potential value for this linker, which influences the formation of the Cu_6_ cluster node similarly to previous cases (Fig. S9†). Interestingly, despite the enhanced N-coordination site on the linker molecule compared to earlier examples, fewer linker molecules were attached to the cluster node—a phenomenon not observed with tridentate linker molecules (Fig. 5A). Due to this reduction of the number of the linker molecule on each cluster node further initiate the coordination of the CF_3_COO^−^ units to the cluster node which ultimately neutralize each cluster node. However, due to the structural differences in the linker molecules, we observed a significant change in both the inter-cluster and interlayer distances. In this structure, the cluster nodes are separated by 8.553 Å along the *X*-axis and 13.941 Å along the *Y*-axis. Although the structural architecture of the framework exhibits a 2D geometry (Fig. 5B) but the closer look infers the ladder-like (Fig. 5C) geometrical pattern due to the non-linear nature of the linker molecule. The interlayer stacking follows an AB stacking mode, with an interlayer spacing of 9.978 Å. The stability and purity of these CAMs in the bulk phase were evaluated by comparing their powder X-ray diffraction patterns with simulated spectra (Fig. S10†). Thermal stability was assessed through thermogravimetric analysis, revealing that while the thermal stability of the various CAMs varies depending on their structure and associated linker molecules, all are generally stable up to 100 °C (Fig. S11†). Elemental composition was determined using SEM-EDS (Fig. S1–S7†), which was further validated by the XPS survey spectra of representative CAMs: Cu_6_-dpb, Cu_10_-dpb, and Cu_7_-tpbt (Fig. S12†). These three CAMs were selected as representative examples. The binding energy spectra of individual elements confirmed the presence of Cu(i) in these CAMs (Fig. S13†). Fourier Transform Infrared (FT-IR) spectroscopy of the bulk crystal further confirmed the preservation of diverse functional groups on their individual surfaces (Fig. S14†).

The ligand system—whether conjugated or non-conjugated—plays a pivotal role in shaping the electronic structure and optical properties of CAMs. Conjugated ligands enable electron delocalization throughout the ligand framework, enhancing electronic coupling and producing distinct optical responses. In contrast, non-conjugated ligands lack this electron delocalization, limiting interaction with the cluster core and resulting in different optical and electronic profiles. This variation in ligand conjugation is thus essential for tuning the UV-visible absorption characteristics of these materials. However, changes in the cluster node and the resulting framework structure can significantly impact the UV-visible absorption properties of CAMs, making it intriguing to analyze this property when multiple factors interplay.

**Fig. 6:**
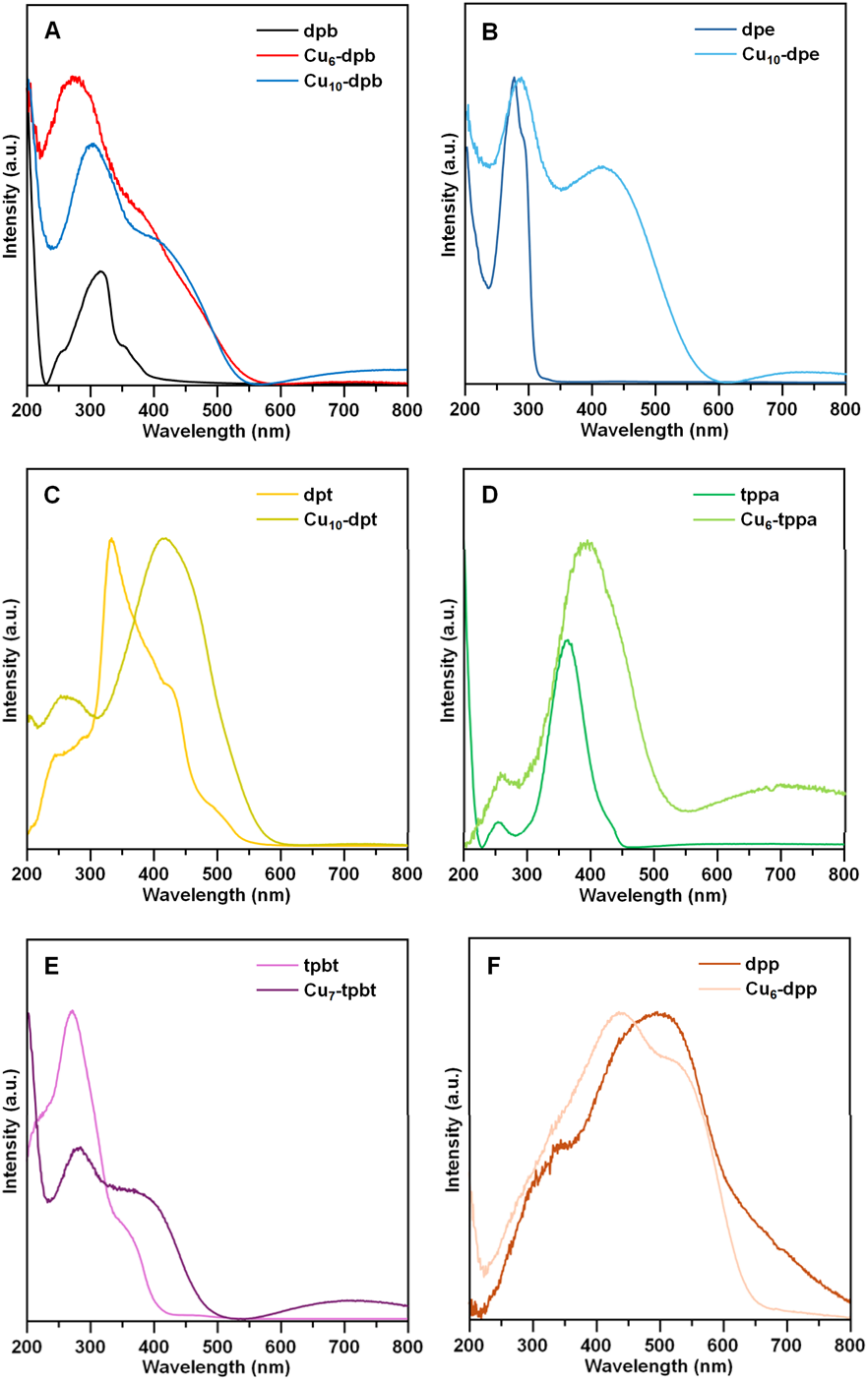
Solid-state UV-vis absorption spectra of (A) dpb, Cu_6_-dpb, Cu_10_-dpb, (B) dpe, Cu_10_-dpe, (C) dpt, Cu_10_-dpt, (D) tppa, Cu_6_-tppa, (E) tpbt, Cu_7_-tpbt, and (F) dpp, Cu_6_-dpp at room temperature.

To investigate this phenomenon, we analyzed the solid-state UV-vis diffuse reflectance spectra of each Cu CAM along with their individual linker molecules. A notable comparison is also evident between the absorbance spectra of the individual CAMs and the precursor [CuS^*t*^Bu]_*n*_ complex (Fig. 6 and S15†). The dpb linker molecule exhibited a broad absorption band in the 230–450 nm range (Fig. 6A), likely due to its π–π* transition.^32,37^ However, after incorporating the linker with the cluster node to form a 2D geometric structure, we observed a significant broadening of the absorption band accompanied by a blue shift in the maximum absorbance wavelength (dpb: 309 nm, Cu_6_-dpb: 275 nm, Cu_10_-dpb: 297 nm) (Fig. 6A). This blue shift becomes more pronounced as the cluster node size decreases. With the broadening of the absorption band, a distinct shoulder peak emerges in each case, likely corresponding to linker-to-metal charge transfer. Notably, a slight red shift in this shoulder peak is observed for the larger cluster node compared to the smaller one (Cu_6_-dpb: 384 nm, Cu_10_-dpb: 414 nm). To gain deeper insights, time-dependent density functional theory (TD-DFT) calculations were performed optimized fragments of Cu_6_-dpb and Cu_10_-dpb NCs were utilized (Fig. S16†). The simulated UV-vis absorption spectra closely align with experimental results, particularly at longer wavelengths where cluster contributions are more pronounced (Fig. S17†). The calculations revealed that the transition at 435 nm for Cu_6_-dpb corresponds to HOMO−4 to LUMO+1, while the transition at 480 nm for Cu_10_-dpb corresponds to HOMO−3 to LUMO+1 (Fig. S18†). Notably, the HOMO is primarily localized on the cluster nodes, whereas the LUMO is predominantly situated on the linker molecules. This suggests that the charge transfer occurs from the cluster nodes to the linker molecules. Similar absorption patterns are observed in Cu_10_-dpb, Cu_10_-dpe, and Cu_10_-dpt when varying the bidentate linker molecules while keeping the cluster node size and overall framework geometry fixed (Fig. 6A–C). Incorporating tridentate linker molecules (tppa and tpbt) results in a significantly broadened absorption profile, independent of the linkers' own absorption characteristics, likely due to the 3D architecture of the resulting structures (Fig. 6D and E). When switching to tetradentate linker molecules, the 2D framework tends to maintain a similar absorption profile relative to the cluster node size (Fig. 6F). However, specific shifts in the maximum absorbance wavelength are observed, reflecting the unique properties of the associated linker molecules. The emission characteristics of these CAMs are also closely tied to those of their individual linker molecules. Since dpb and tppa linker molecules exhibit emission properties, the CAMs synthesized with these linkers also display similar behavior (Fig. S19†). Notably, a significant red shift in the emission maximum occurs upon coordination with the cluster nodes. However, altering the cluster nodes does not significantly affect the emission wavelength, which is predominantly governed by the structure of specific linker molecules.

## Conclusions

In conclusion, we have successfully developed a straightforward one-pot reaction method to synthesize a variety of Cu CAMs through a liquid–liquid interface crystallization technique. Our strategic exploration reveals several key factors that influence the formation and properties of Cu CAMs:

Electron-donating environment of linkers: the electron-donating nature of the linker molecules plays a crucial role in determining the size and geometry of the cluster nodes. Linkers with stronger electron-donating capabilities tend to produce more bigger cluster size.

Solvent medium effects: the choice of solvent exerts a significant influence on the electronic environment of the linker molecules, affecting the cluster node size determination. Changes in solvent polarity and compatibility alter the electronic properties of the linkers, which, in turn, impact the structural features of the resulting cluster nodes and framework.

Linker coordination sites: the number and positioning of coordinating sites on the linker molecules are essential in guiding the surface architecture of the cluster nodes and the overall framework geometry. These coordinating sites act as anchors, dictating the specific attachment points for cluster assembly and thus shaping the final arrangement.

Geometric architecture of linkers: the intrinsic geometry of the linker molecules themselves—whether linear or non-linear—significantly impacts the final framework structure.

The diverse frameworks obtained by integrating various linker molecules exhibit unique photophysical properties, which are closely linked to the electronic and structural characteristics of the linkers and the resulting frameworks. These findings provide a valuable foundation for future research, offering guidance for the design and synthesis of novel Cu CAMs with precise structural architectures. Additionally, it highlights strategies for tailoring the photophysical properties of Cu CAMs to suit specific applications.

## Data availability

The ESI† contains the crystallographic parameters, SEM images and corresponding EDX elemental maps, PXRD, TGA, and FT-IR of Cu_6_-dpb, Cu_10_-dpb, Cu_10_-dpe, Cu_10_-dpt, Cu_6_-tppa, Cu_7_-tpbt and Cu_6_-dpp CAMs, XPS of Cu_6_-dpb, Cu_10_-dpb, Cu_7_-tpbt and zeta potential of all linkers at pH 7.0. Crystallographic data for Cu_6_-dpb, Cu_10_-dpb, Cu_10_-dpe, Cu_10_-dpt, Cu_6_-tppa, Cu_7_-tpbt and Cu_6_-dpp CAMs have been deposited at the CCDC under CCDC numbers 2400572, 2400576, 2400577, 2400578, 2400574, 2400575 and 2400573, respectively.

## Author contributions

Saikat Das and Y. N. conceived the research and supervised the project. J. S., R. N. and A. K. performed the syntheses and characterizations. R. T., T. K., Subhabrata Das and S. T. helped in the experiments. S. B. undertook the data analysis and wrote the manuscript.

## Conflicts of interest

There are no conflicts to declare.

## Supplementary Material

SC-OLF-D4SC07730J-s001

SC-OLF-D4SC07730J-s002
