# Supplementary material for: Tunable structural rearrangement in Cu cluster assemblies through linker and solvent alterations
Source: Chem Sci. 2025 Jan 22;16(6):2600–8. doi: 10.1039/d4sc07730j (PMC11775578; doi:10.1039/d4sc07730j)
Supplement: SC-016-D4SC07730J-s001 [file SC-016-D4SC07730J-s001.pdf]

Supplementary Information (SI) for Chemical Science.
This journal is © The Royal Society of Chemistry 2025

Supporting Information for

Tunable structural rearrangement in Cu cluster assemblies through linker and solvent alterations

Saikat Das,[a] *Jin Sakai,*[b] *Riki Nakatani,*[b] *Ayumu Kondo,*[b] *Rina Tomioka,*[b] *Subhabrata Das,*[c] *Shuntaro Takahashi,*[c] *Tokuhisa Kawawaki,*[b] *Sourav Biswas,**[a] *and Yuichi Negishi**[a,d]

[a]Research Institute for Science & Technology, Tokyo University of Science, Tokyo 162-8601, Japan.

[b]Department of Applied Chemistry, Faculty of Science, Tokyo University of Science, Kagurazaka, Shinjuku-ku, Tokyo 162-8601, Japan.

[c]Chemical Materials Development Department, Tanaka Kikinzoku Kogyo K.K., Tsukuba Technical Center, 22 Wadai, Tsukuba, Ibaraki 300-4247 (Japan).

[d]Institute of Multidisciplinary Research for Advanced Materials, Tohoku University, Aoba-ku, Sendai 980-8577, Japan.

*Correspondence to: sourav.biswas210@gmail.com (S.B.), yuichi.negishi.a8@tohoku.ac.jp (Y.N.)

Table of Contents

Name	Description	Page No.
	General information	S3
Table S1	Crystallographic parameters of **Cu_6-dpb** CAM	S5
Table S2	Crystallographic parameters of **Cu_{10}-dpb** CAM	S6
Table S3	Crystallographic parameters of **Cu_{10}-dpe** CAM	S7
Table S4	Crystallographic parameters of **Cu_{10}-dpt** CAM	S8
Table S5	Crystallographic parameters of **Cu_6-tppa** CAM	S9
Table S6	Crystallographic parameters of **Cu_7-tpbt** CAM	S10
Table S7	Crystallographic parameters of **Cu_6-dpp** CAM	S11
Table S8	Bond length and bond angle comparison	S12
Fig. S1	SEM image and corresponding EDX elemental maps of **Cu_6-dpb** CAM	S13
Fig. S2	SEM image and corresponding EDX elemental maps of **Cu_{10}-dpb** CAM	S13
Fig. S3	SEM image and corresponding EDX elemental maps of **Cu_{10}-dpe** CAM	S13
Fig. S4	SEM image and corresponding EDX elemental maps of **Cu_{10}-dpt** CAM	S13
Fig. S5	SEM image and corresponding EDX elemental maps of **Cu_6-tppa** CAM	S13
Fig. S6	SEM image and corresponding EDX elemental maps of **Cu_7-tpbt** CAM	S14
Fig. S7	SEM image and corresponding EDX elemental maps of **Cu_6-dpp** CAM	S14
Fig. S8	Zeta potential of dpb, dpe and dpt linkers at pH 7.0	S14
Fig. S9	Zeta potential of tppa, tpbt, dpp linkers at pH 7.0	S15
Fig. S10	PXRD patterns of individual Cu CAMs	S15
Fig. S11	TGA curves of all Cu CAMs	S16
Fig. S12	XPS survey spectra of three representative Cu CAMs	S16
Fig. S13	XPS binding energy of Cu, S and C for representative Cu CAMs	S17
Fig. S14	FT-IR spectra of all Cu CAMs	S18
Fig. S15	Solid-state UV-vis absorption spectra of $[CuS^tBu]_n$ complex	S18
Fig. S16	Optimized fragmented structure of representative Cu CAMs	S19
Fig. S17	Simulated UV-vis absorbance spectra of representative Cu CAMs	S19
Fig. S18	Orbital contribution of the electronic transitions	S20
Fig. S19	Emission properties of Cu CAMs and their linker molecules	S20
	References	S21

General information

Materials

Unless specified otherwise, all reagents and solvents were procured from commercial sources and used directly as received. Copper(II) nitrate trihydrate ($Cu(NO_3)_2 \cdot 3H_2O$)), triethylamine (NEt_3), trifluoroacetic acid (CF_3COOH), phosphate buffer solution (pH 7.0), and methanol (MeOH) were obtained from FUJIFILM Wako Pure Chemical Corporation. Acetonitrile (MeCN), dimethylacetamide (DMAc), dichloromethane (DCM), *N*-Methyl-2-pyrrolidone (NMP), and chloroform ($CHCl_3$) were obtained from Kanto Chemical Co., Inc. *tert*-butyl mercaptan was obtained from Tokyo Chemical Industry Co., Ltd. 1,4-Di(4-pyridyl)benzene) (dpb), 1,2-Di(pyridin-4-yl)ethyne (dpe), 5,5'-Di(pyridin-4-yl)-2,2'-bithiophene (dpt), Tris(4-(pyridin-4-yl)phenyl)amine (tppa), N1,N3,N5-Tri(pyridin-4-yl)benzene-1,3,5-tricarboxamide (tpbt), and 3,3'-[2-(4,5-Di-3-pyridinyl-1,3-dithiol-2-ylidene)-1,3-dithiole-4,5-diyl]bis[pyridine] (dpp) were obtained from ET Co., Ltd.

Instrumentation

For the single-crystal X-ray diffraction (SCXRD) data collection, the single crystals were first immersed in cryoprotectant Parabar 10312 (Hampton Research, 34 Journey, Aliso Viejo, CA 92656-3317 USA) followed by mounting on a Dual-Thickness MicroMounts™ (MiTeGen, LLC, Ithaca, NY, USA). The diffraction data for the single crystals were collected from a Bruker D8 QUEST and a XtaLAB Synergy-DW SCXRD diffractometers equipped with monochromatic Mo Kα radiation (λ = 0.71073 Å) and Cu Kα radiation (λ = 1.5418 Å), respectively. The crystal structures were determined by utilizing the Apex5 Bruker software[1] and CrysAlis^{Pro} software[2]. The optical microscope images were obtained with an Olympus SZX7 stereo microscope. Scanning electron microscopy/energy-dispersive X-ray spectroscopy (SEM-EDX) analysis were carried out using a JEOL JSM-7001F/SHL field emission scanning electron microscope. Diffuse reflectance spectroscopy (DRS) data were collected on a JASCO V-670 spectrophotometer. The zeta potential measurements were performed on a Malvern Panalytical Zetasizer Nano ZS size analyzer.

DFT calculations

All geometry optimizations and time-dependent density functional theory (TD-DFT) calculations were carried out with the CP2K program[3], utilizing the Gaussian and plane-wave (GPW) formalism. Valence electrons were treated explicitly, and norm-conserving Goedecker–Teter–Hutter (GTH) pseudopotentials[4] were used to account for the interactions between the valence electrons and the atomic cores. We employed DZVP-MOLOPT-SR-GTH basis sets, and a cutoff of 600 Ry for the auxiliary plane-wave basis set. Kohn–Sham density functional theory (KS-DFT)[5,6] simulations were carried out employing the Perdew–Burke–Ernzerhof (PBE)[7] exchange–correlation functional supplemented with the Grimmes D3BJ[8] dispersion correction for geometry optimization. The TD-DFT calculations were performed at the level of PBE0/DZVP-MOLOPT-SR-GTH. The wavefunction analyses were carried out using Multiwfn 3.8(dev). Structural visualization was performed using VESTA 3.5.7 software.

Table S1. Crystallographic parameters of **Cu_6-dpb**.

Identification code	**Cu_6-dpb**
Empirical formula	$C_{82}H_{86}Cu_6F_9N_7O_7S_4$
CCDC number	2400572
Formula weight	1888.83
Temperature/K	293 K
Crystal system	Orthorhombic
Space group	*Pnma*
a/Å	36.1356(2)
b/Å	16.4719(2)
c/Å	28.0985(3)
α/°	90
β/°	90
γ/°	90
Volume/Å^3	16724.8(3)
Z	8
ρ_{calc}/g cm^{-3}	1.500
μ/mm^{-1}	3.796
F(000)	7712
Radiation	CuKα (λ = 1.54184)
2Θ range for data collection/°	3.342 to 71.217°
Index ranges	$-44\leq h\leq 16$, $-14\leq k\leq 20$, $-33\leq l\leq 34$
Reflections collected	51292
Independent reflections	16378 [R_{int} = 0.0322]
Data/restraints/parameters	16378/964/771
Goodness-of-fit on F^2	1.055
Final R indexes [I>=2σ (I)]	R_1 = 0.1032, wR_2 = 0.3038
Final R indexes [all data]	R_1 = 0.1215, wR_2 = 0.3217
Largest diff. peak/hole / e Å^{-3}	2.483 / -1.483

Table S2. Crystallographic parameters of **Cu_{10}-dpb**.

Identification code	**Cu_{10}-dpb**
Empirical formula	$C_{13.7}H_{3.97}Cu_{0.48}F_{0.571}N_{0.19}O_{0.38}S_{0.29}$
CCDC number	2400576
Formula weight	118.92
Temperature/K	273
Crystal system	Triclinic
Space group	$P\bar{1}$
a/Å	13.4577(14)
b/Å	13.9427(13)
c/Å	13.9994(14)
α/°	82.041(3)
β/°	82.058(3)
γ/°	75.543(3)
Volume/$Å^3$	2504.4(4)
Z	21
ρ_{calc}/g cm^{-3}	1.656
μ/mm^{-1}	2.545
F(000)	1248
Radiation	MoKα (λ = 0.71073)
2Θ range for data collection/°	2.000 to 22.212°
Index ranges	-14≤h≤14, -14≤k≤14, -14≤l≤14
Reflections collected	16723
Independent reflections	6182 [R_{int} = 0.0596]
Data/restraints/parameters	6182/7/478
Goodness-of-fit on F^2	1.024
Final R indexes [I>=2σ (I)]	R_1 = 0.0681, wR_2 = 0.1717
Final R indexes [all data]	R_1 = 0.0981, wR_2 = 0.1916
Largest diff. peak/hole / e $Å^{-3}$	1.904 / -1.857

Table S3. Crystallographic parameters of **Cu_{10}-dpe**.

Identification code	**Cu_{10}-dpe**
Empirical formula	$C_{39}H_{37}Cu_5F_6N_4O_4S_3$
CCDC number	2400577
Formula weight	1080.38
Temperature/K	273
Crystal system	Monoclinic
Space group	$P2_1/c$
a/Å	13.9234(4)
b/Å	11.9576(4)
c/Å	23.9446(7)
α/°	90
β/°	91.9010(10)
γ/°	90
Volume/Å^3	3984.4(2)
Z	4
ρ_{calc}/g cm^{-3}	1.801
μ/mm^{-1}	2.982
F(000)	2160
Radiation	MoKα (λ = 0.71073)
2Θ range for data collection/°	2.208 to 25.350°
Index ranges	$-16\leq h\leq 16$, $-14\leq k\leq 14$, $-23\leq l\leq 28$
Reflections collected	28226
Independent reflections	7234 [R_{int} = 0.0481]
Data/restraints/parameters	7234/391/469
Goodness-of-fit on F^2	0.997
Final R indexes [I>=2σ (I)]	R_1 = 0.0513, wR_2 = 0.1023
Final R indexes [all data]	R_1 = 0.0697, wR_2 = 0.1100
Largest diff. peak/hole / e Å^{-3}	1.904 / -1.857

Table S4. Crystallographic parameters of **Cu_{10}-dpt**.

Identification code	**Cu_{10}-dpt**
Empirical formula	$C_{12.96}H_{3.28}Cu_{0.40}F_{0.48}N_{0.16}O_{0.32}S_{0.40}$
CCDC number	2400578
Formula weight	97.33
Temperature/K	90
Crystal system	Monoclinic
Space group	$P2_1/c$
a/Å	20.5359(10)
b/Å	15.9060(8)
c/Å	18.1932(10)
α/°	90
β/°	102.802(2)
γ/°	90
Volume/Å^3	5795.0(5)
Z	14
ρ_{calc}/g cm^{-3}	1.394
μ/mm^{-1}	2.128
F(000)	2440
Radiation	MoKα (λ = 0.71073)
2Θ range for data collection/°	2.123 to 26.638°
Index ranges	$-25\leq h\leq 25$, $-19\leq k\leq 20$, $-22\leq l\leq 22$
Reflections collected	58049
Independent reflections	11971 [R_{int} = 0.0741]
Data/restraints/parameters	11971/39/544
Goodness-of-fit on F^2	1.050
Final R indexes [I>=2σ (I)]	R_1 = 0.0582, wR_2 = 0.1524
Final R indexes [all data]	R_1 = 0.0958, wR_2 = 0.1738
Largest diff. peak/hole / e Å^{-3}	1.011 / -1.024

Table S5. Crystallographic parameters of **Cu_6-tppa**.

Identification code	**Cu_6-tppa**
Empirical formula	$C_{124}H_{178.5}Cu_6N_{18.5}O_{10.5}S_4$
CCDC number	2400574
Formula weight	2605.82
Temperature/K	293
Crystal system	Cubic
Space group	$Pa\bar{3}$
a/Å	30.9370(4)
b/Å	30.9370(4)
c/Å	30.9370(4)
α/°	90
β/°	90
γ/°	90
Volume/Å^3	29609.8(10)
Z	8
ρ_{calc}/g cm^{-3}	1.169
μ/mm^{-1}	1.901
F(000)	10992
Radiation	CuKα (λ = 1.54184)
2Θ range for data collection/°	4.287 to 63.818°
Index ranges	$-33\leq h\leq 27$, $-26\leq k\leq 34$, $-25\leq l\leq 36$
Reflections collected	20402
Independent reflections	7963 [R_{int} = 0.0321]
Data/restraints/parameters	7963/305/256
Goodness-of-fit on F^2	1.028
Final R indexes [I>=2σ (I)]	R_1 = 0.1048, wR_2 = 0.1627
Final R indexes [all data]	R_1 = 0.2063, wR_2 = 0.2430
Largest diff. peak/hole / e Å^{-3}	0.298 / -0.206

Table S6. Crystallographic parameters of **Cu_7-tpbt**.

Identification code	**Cu_7-tpbt**
Empirical formula	$C_{82}H_{99}Cu_7F_9N_{15}O_{15}S_4$
CCDC number	2400575
Formula weight	2278.78
Temperature/K	273
Crystal system	Monoclinic
Space group	*C*2/*c*
a/Å	40.186(2)
b/Å	17.9336(10)
c/Å	43.303(2)
α/°	90
β/°	108.171(2)
γ/°	90
Volume/Å^3	29561(3)
Z	8
ρ_{calc}/g cm^{-3}	1.021
μ/mm^{-1}	1.096
F(000)	9312
Radiation	MoKα (λ = 0.71073)
2Θ range for data collection/°	1.898 to 25.090°
Index ranges	$-47\leq h\leq 47$, $-21\leq k\leq 21$, $-51\leq l\leq 51$
Reflections collected	163726
Independent reflections	26252 [R_{int} = 0.1258]
Data/restraints/parameters	26252/954/1027
Goodness-of-fit on F^2	1.080
Final R indexes [I>=2σ (I)]	R_1 = 0.0791, wR_2 = 0.2368
Final R indexes [all data]	R_1 = 0.1198, wR_2 = 0.2649
Largest diff. peak/hole / e Å^{-3}	2.301 / -1.104

Table S7. Crystallographic parameters of **Cu$_6$-dpp**.

Identification code	**Cu$_6$-dpp**
Empirical formula	$C_{57.5}H_{48}Cu_6F_3N_4O_2S_8$
CCDC number	2400573
Formula weight	1518.82
Temperature/K	273.15
Crystal system	Orthorhombic
Space group	*Imma*
a/Å	24.6205(9)
b/Å	17.0084(7)
c/Å	22.4482(9)
α/°	90
β/°	90
γ/°	90
Volume/Å^3	9400.3(6)
Z	4
ρ_{calc}/g cm^{-3}	1.073
μ/mm^{-1}	1.683
F(000)	3044
Radiation	MoKα (λ = 0.71073)
2Θ range for data collection/°	2.326 to 25.672°
Index ranges	$-30 \leq h \leq 30$, $-20 \leq k \leq 20$, $-27 \leq l \leq 27$
Reflections collected	48189
Independent reflections	4702 [R_{int} = 0.0681]
Data/restraints/parameters	4702/389/282
Goodness-of-fit on F^2	1.089
Final R indexes [I>=2σ (I)]	R_1 = 0.0796, wR_2 = 0.1768
Final R indexes [all data]	R_1 = 0.0961, wR_2 = 0.1862
Largest diff. peak/hole / e Å^{-3}	2.045 / -0.858

Table S8. Bond length and bond angles of (A) **Cu_6-dpb** and (C) **Cu_{10}-dpb** cluster nodes, compared with the (B) Cu_6 nanoclusters[9] and (D) Cu_{10} nanoclusters[10] reported in literature.

(A)

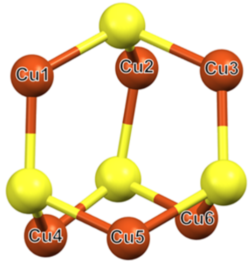

Atom1	Atom2	Bond length/Å		/Å
Cu1	Cu2	3.691	Maximum	3.851
Cu1	Cu3	3.691	Minimum	2.953
Cu1	Cu4	3.734	Average	3.464
Cu1	Cu5	3.734	S.D.	0.3202
Cu2	Cu3	3.248		
Cu2	Cu4	3.851		
Cu2	Cu6	3.347		
Cu3	Cu5	3.851		
Cu3	Cu6	3.347		
Cu4	Cu5	3.171		
Cu4	Cu6	2.953		
Cu5	Cu6	2.953		

Atoms	Angle/°
Cu1-Cu2-Cu3	63.90
Cu1-Cu3-Cu2	63.90
Cu2-Cu1-Cu3	52.21
Cu1-Cu2-Cu4	59.31
Cu1-Cu4-Cu2	58.22
Cu2-Cu1-Cu4	62.47
Cu2-Cu3-Cu6	60.97
Cu2-Cu6-Cu3	58.06
Cu3-Cu2-Cu6	60.97
Cu4-Cu5-Cu6	57.52
Cu4-Cu6-Cu5	64.96
Cu5-Cu4-Cu6	57.52

(B)

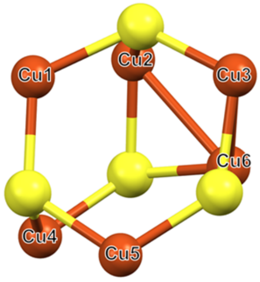

Atom1	Atom2	Bond length/Å		/Å
Cu1	Cu2	2.895	Maximum	4.07
Cu1	Cu3	3.972	Minimum	2.862
Cu1	Cu4	3.527	Average	3.521
Cu1	Cu5	3.922	S.D.	0.4312
Cu2	Cu3	3.651		
Cu2	Cu4	3.840		
Cu2	Cu6	2.862		
Cu3	Cu5	4.070		
Cu3	Cu6	2.883		
Cu4	Cu5	3.209		
Cu4	Cu6	3.860		
Cu5	Cu6	3.565		

Atoms	Angle/°
Cu1-Cu2-Cu3	73.70
Cu1-Cu3-Cu2	44.40
Cu2-Cu1-Cu3	61.90
Cu1-Cu2-Cu4	61.26
Cu1-Cu4-Cu2	46.05
Cu2-Cu1-Cu4	72.69
Cu2-Cu3-Cu6	50.30
Cu2-Cu6-Cu3	78.91
Cu3-Cu2-Cu6	50.80
Cu4-Cu5-Cu6	69.25
Cu4-Cu6-Cu5	51.03
Cu5-Cu4-Cu6	59.72

(C)

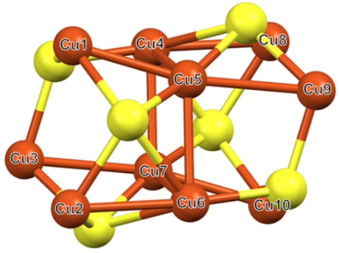

Atom1	Atom2	Bond length/Å		/Å
Cu1	Cu2	3.577	Maximum	3.577
Cu1	Cu3	3.523	Minimum	2.602
Cu1	Cu5	2.602	Average	3.007
Cu2	Cu3	3.428	S.D.	0.3946
Cu2	Cu6	2.688		
Cu3	Cu7	2.778		
Cu4	Cu5	2.793		
Cu4	Cu7	2.67		
Cu4	Cu8	2.688		
Cu5	Cu6	2.67		
Cu5	Cu9	2.778		
Cu6	Cu7	2.793		
Cu7	Cu10	2.602		
Cu8	Cu9	3.428		
Cu8	Cu10	3.577		
Cu9	Cu10	3.523		

Atoms	Angle/°	Atoms	Angle/°
Cu1-Cu2-Cu3	60.35	Cu5-Cu4-Cu7	66.92
Cu1-Cu3-Cu2	61.91	Cu5-Cu6-Cu7	66.92
Cu2-Cu1-Cu3	57.73	Cu4-Cu5-Cu9	101.37
Cu1-Cu4-Cu5	47.44	Cu4-Cu8-Cu9	88.76
Cu1-Cu5-Cu4	80.30	Cu5-Cu4-Cu8	91.69
Cu4-Cu1-Cu5	52.26	Cu5-Cu9-Cu8	77.89
Cu3-Cu2-Cu6	88.76	Cu6-Cu7-Cu10	80.30
Cu3-Cu7-Cu6	101.37	Cu6-Cu10-Cu7	52.26
Cu2-Cu3-Cu7	77.89	Cu7-Cu6-Cu10	44.74
Cu2-Cu6-Cu7	91.69	Cu8-Cu9-Cu10	61.91
Cu4-Cu5-Cu6	113.08	Cu8-Cu10-Cu9	57.73
Cu4-Cu7-Cu6	113.08	Cu9-Cu8-Cu10	60.35

(D)

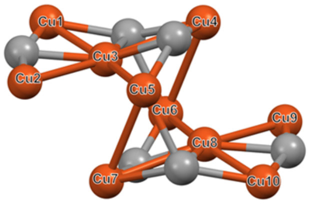

Atom1	Atom2	Bond length/Å		/Å
Cu1	Cu3	2.502	Maximum	2.732
Cu2	Cu3	2.675	Minimum	2.502
Cu3	Cu4	2.703	Average	2.657
Cu3	Cu5	2.732	S.D.	0.0804
Cu4	Cu6	2.671		
Cu5	Cu7	2.671		
Cu6	Cu8	2.732		
Cu7	Cu8	2.703		
Cu8	Cu9	2.675		
Cu8	Cu10	2.502		

Atoms	Angle/°
Cu1-Cu3-Cu2	99.14
Cu1-Cu3-Cu4	95.69
Cu2-Cu3-Cu5	66.16
Cu4-Cu3-Cu5	97.02
Cu3-Cu4-Cu6	67.02
Cu3-Cu5-Cu7	71.77
Cu4-Cu6-Cu8	71.77
Cu5-Cu7-Cu8	67.02
Cu6-Cu8-Cu7	97.02
Cu6-Cu8-Cu9	66.16
Cu7-Cu8-Cu10	95.69
Cu9-Cu8-Cu10	99.14

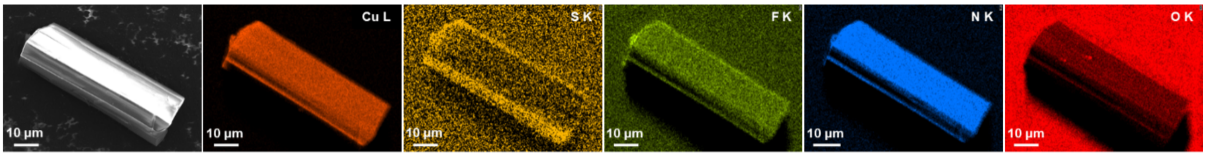

Fig. S1 SEM image and corresponding EDX elemental maps of **Cu_6-dpb** CAM.

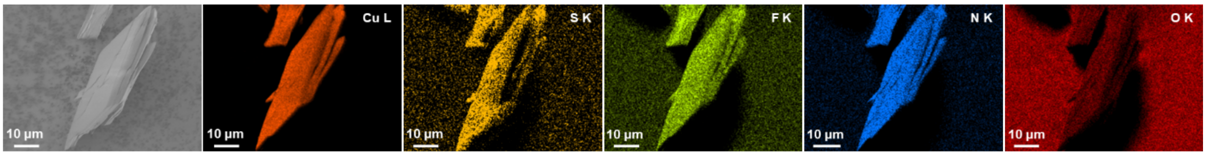

Fig. S2 SEM image and corresponding EDX elemental maps of **Cu_{10}-dpb** CAM.

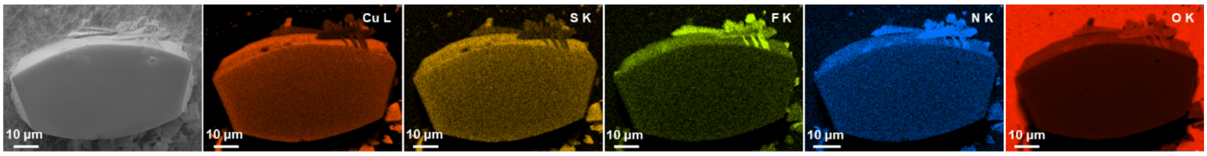

Fig. S3 SEM image and corresponding EDX elemental maps of **Cu_{10}-dpe** CAM.

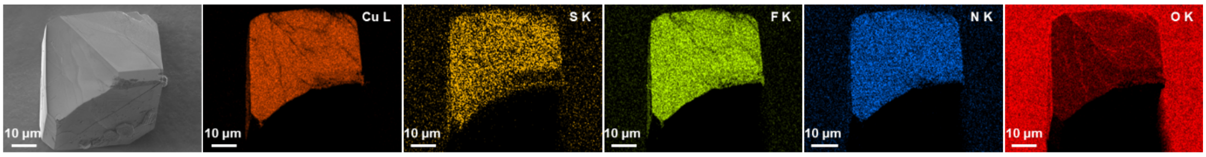

Fig. S4 SEM image and corresponding EDX elemental maps of **Cu_{10}-dpt** CAM.

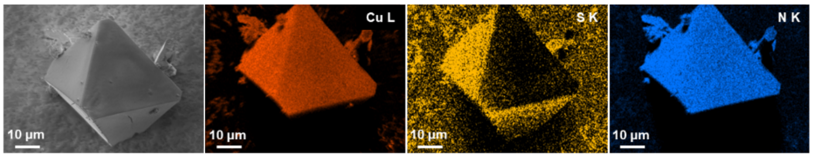

Fig. S5 SEM image and corresponding EDX elemental maps of **Cu_6-tppa** CAM.

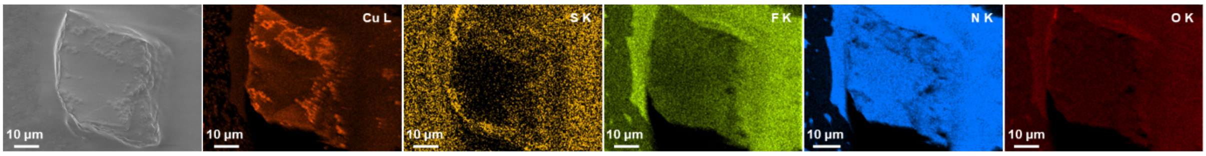

Fig. S6 SEM image and corresponding EDX elemental maps of **Cu_7-tpbt** CAM.

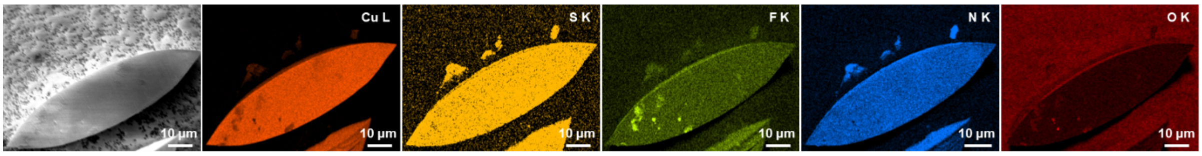

Fig. S7 SEM image and corresponding EDX elemental maps of **Cu_6-dpp** CAM.

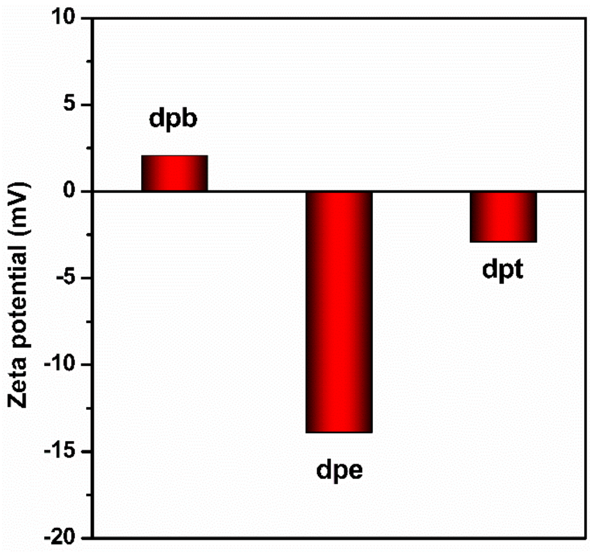

Fig. S8 Zeta potential of dpb, dpe and dpt linkers at pH 7.0.

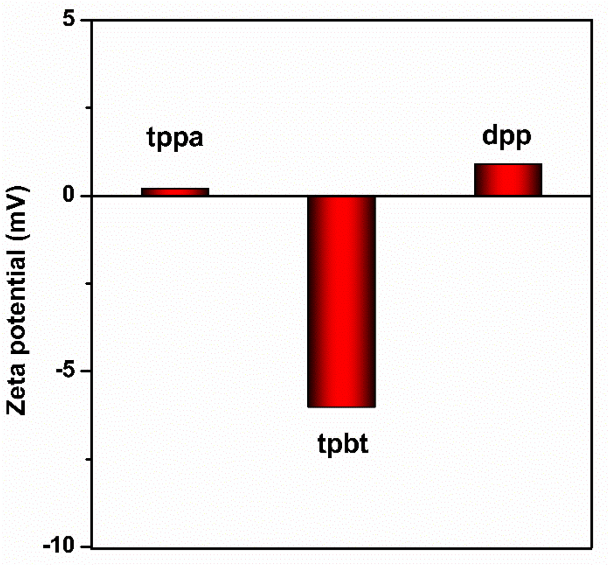

Fig. S9 Zeta potential of tppa, tpbt and dpp linkers at pH 7.0.

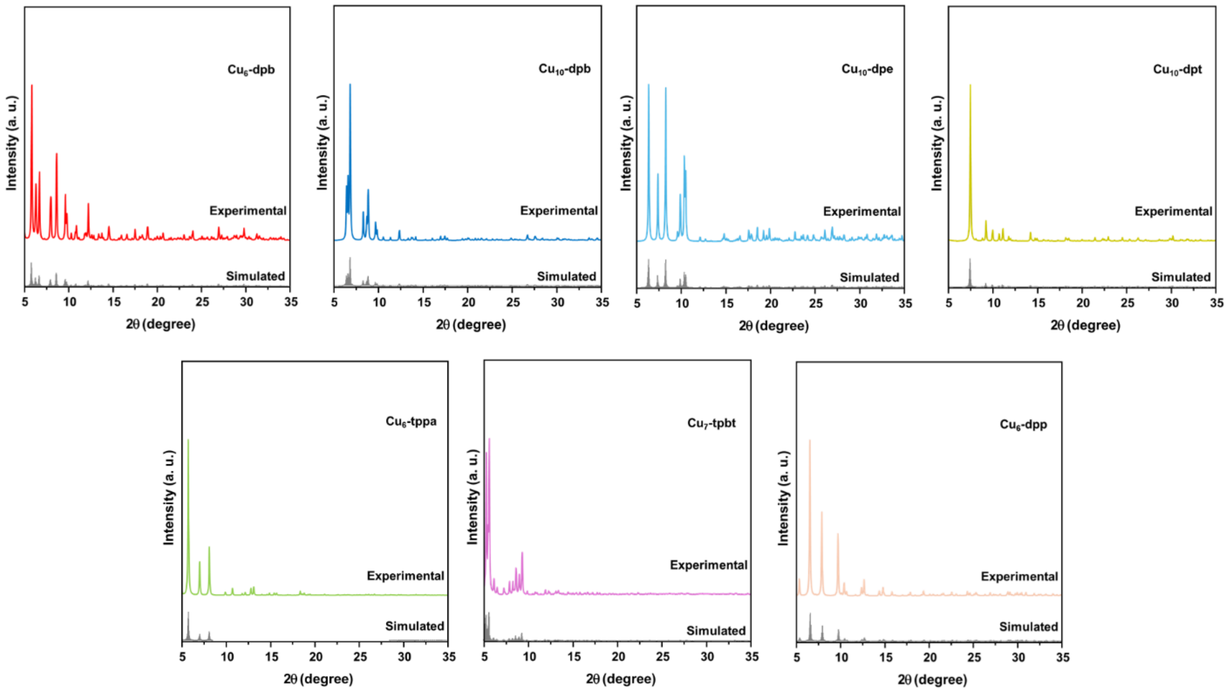

Fig. S10 Matching the experimental PXRD patterns of individual Cu CAMs with their corresponding simulated patterns.

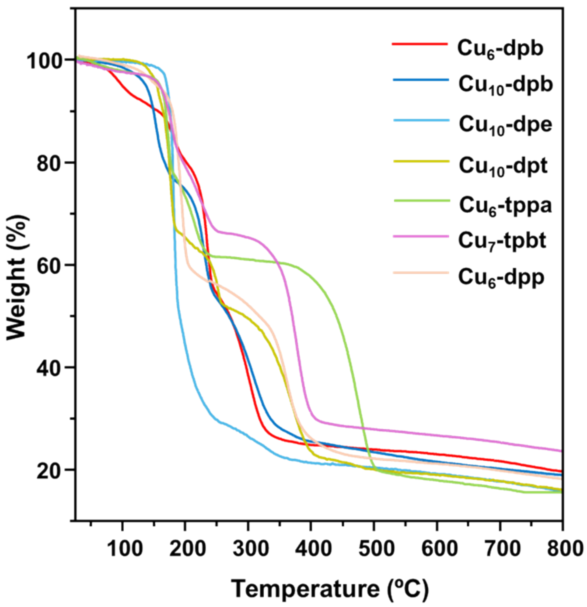

Fig. S11 TGA curves of all Cu CAMs.

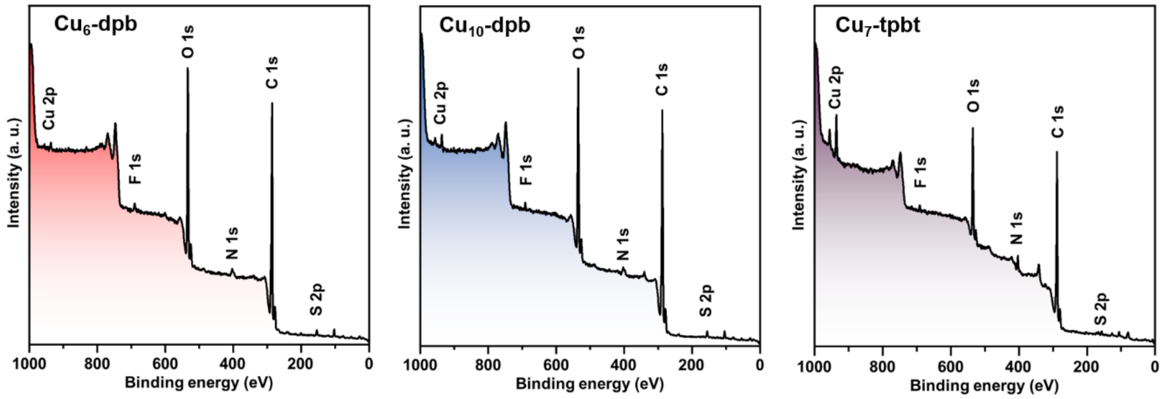

Fig. S12 XPS survey spectra of three representative Cu CAMs.

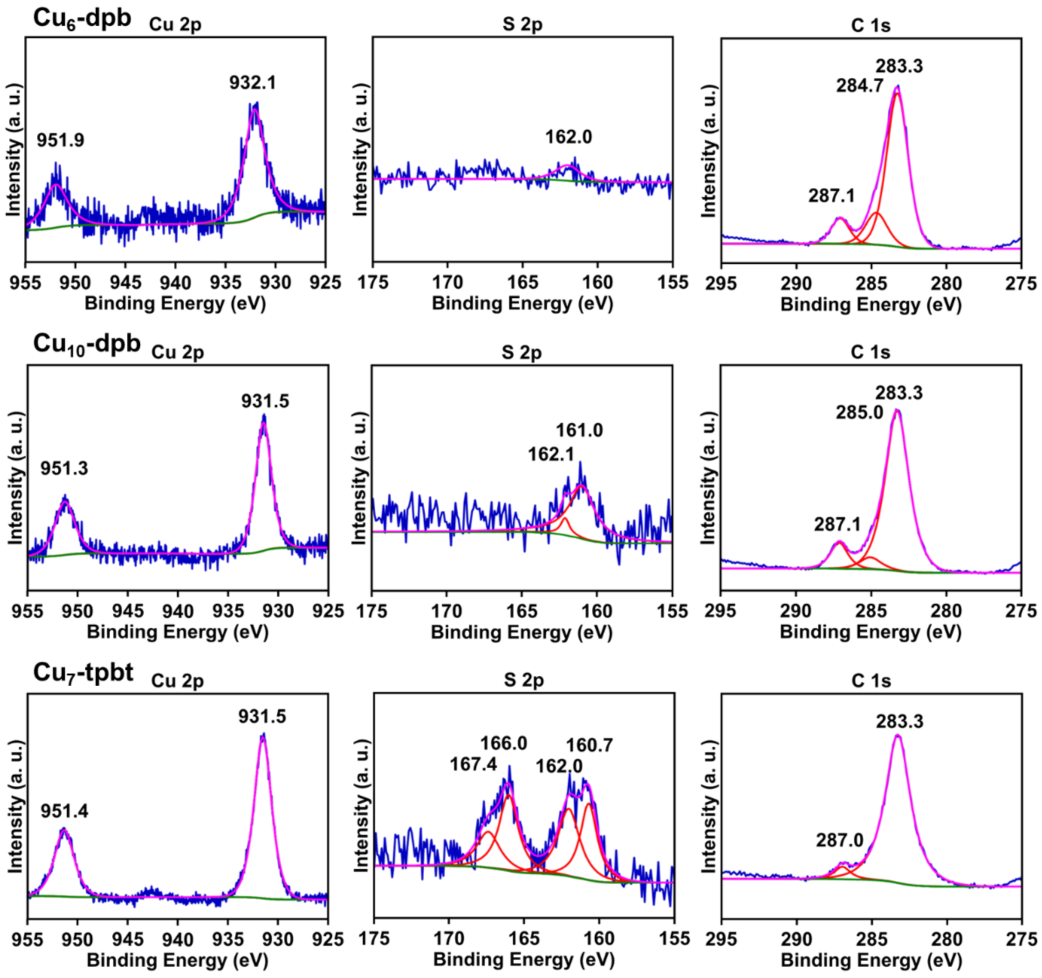

Fig. S13 XPS binding energy of individual Cu, S and C for three Cu CAMs.

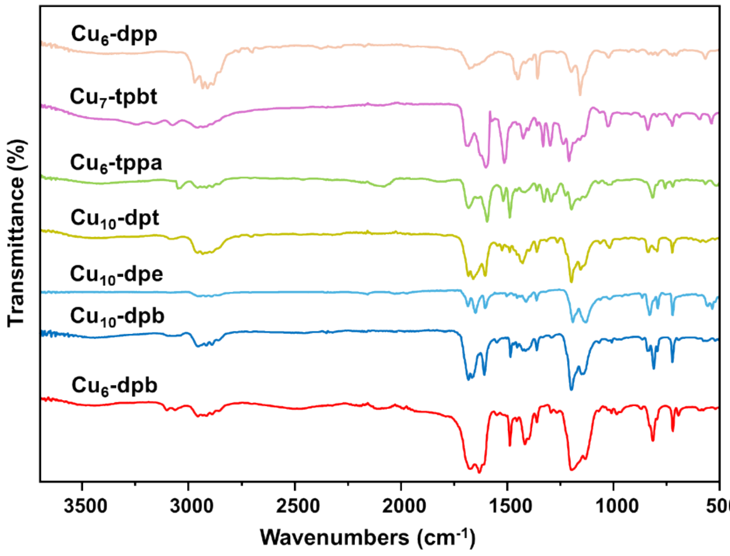

Fig. S14 FT-IR spectra of all Cu CAMs. The multiple peaks from ~3050 to ~2850 cm^{-1} in the spectrum is assigned to the C-H stretching vibration of thiolate ligands, the peak ~1650 cm^{-1} observed in all of the structures corresponds to C═O in the CF_3COO^-, multiple peaks ~1100–1250 cm^{-1} correspond to CF_3 in CF_3COO^-. The peak at 1600 cm^{-1} corresponds to the ring deformation vibration in the pyridine molecular plane, and the multiple peaks at ~700–850 cm^{-1} correspond to C-H in the aromatic ring.

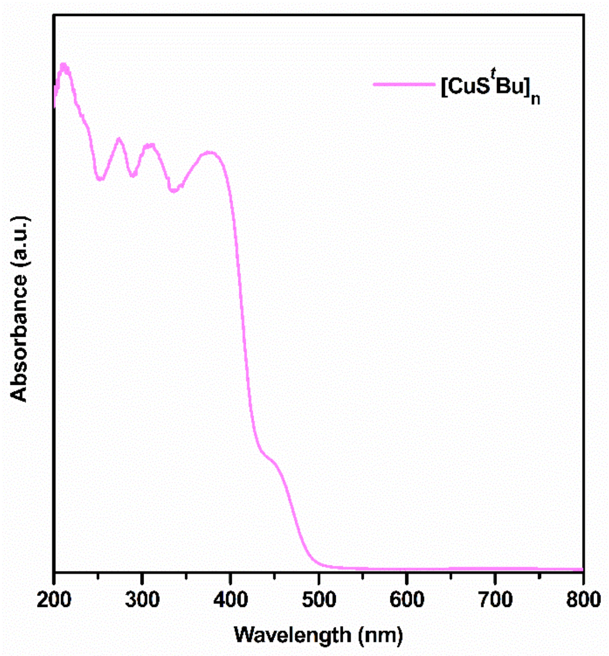

Fig. S15 Solid-state UV-vis absorption spectra of [CuS^{t}Bu]$_n$ complex.

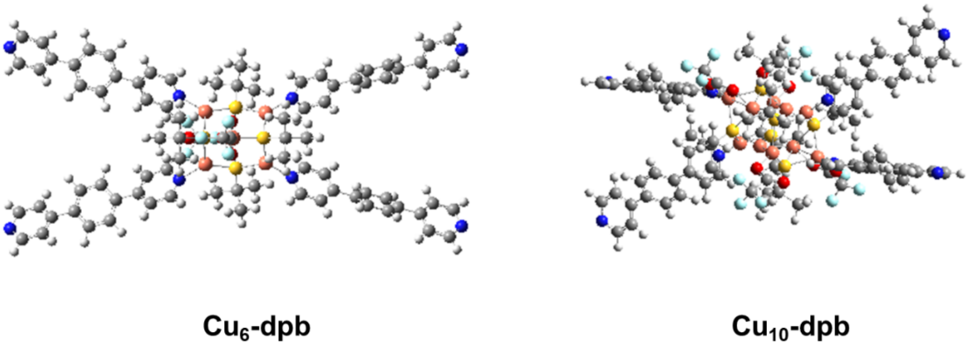

Fig. S16 Optimized fragmented structure of **Cu_6-dpb** and **Cu_{10}-dpb** CAMs.

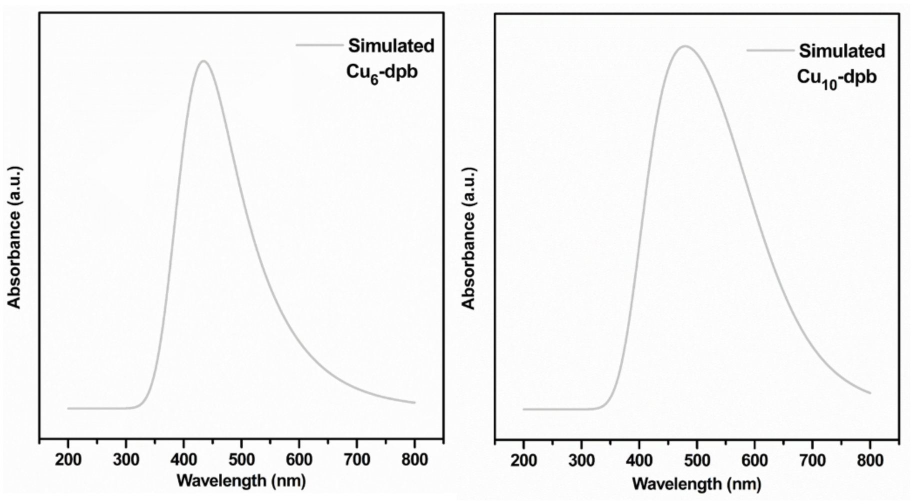

Fig. S17 Simulated UV-vis absorbance spectra of corresponding Cu CAMs.

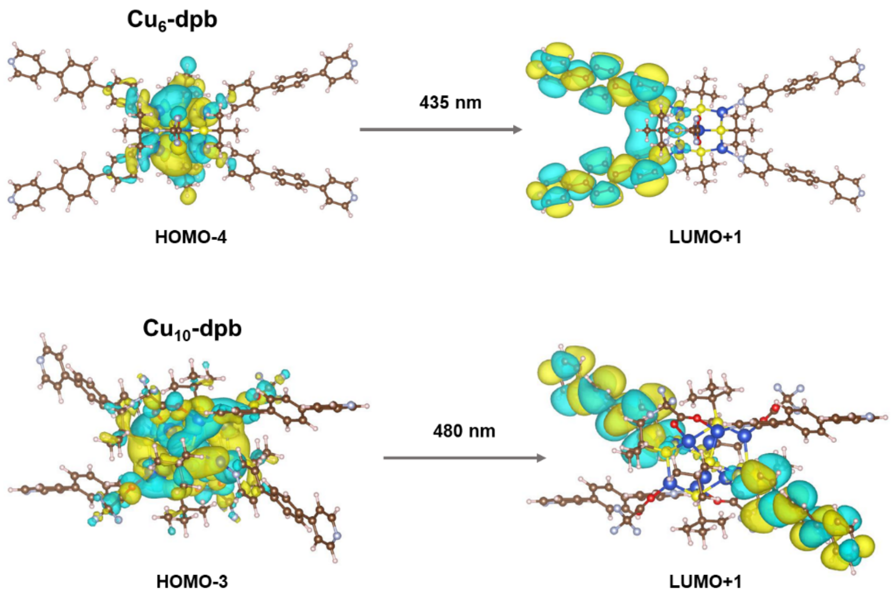

Fig. S18 Orbital contribution of the electronic transitions at their corresponding wavelength.

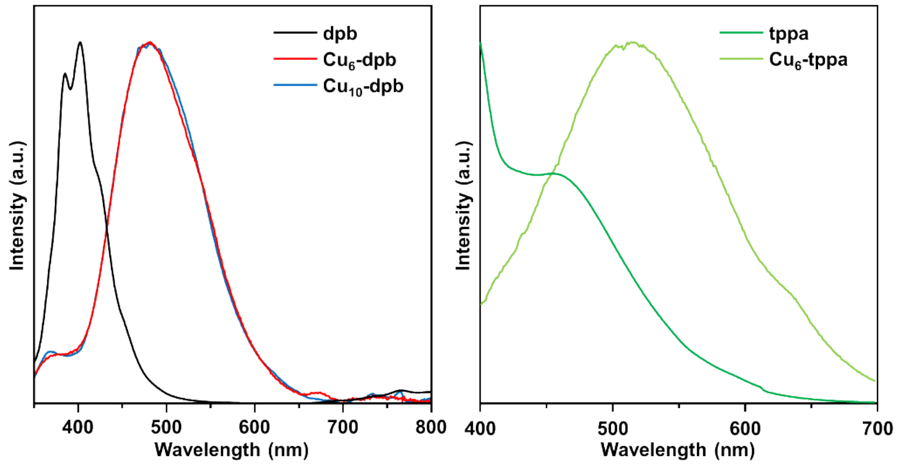

Fig. S19 Emission properties of corresponding Cu CAMs and their linker molecules.

References

1. Bruker APEX5, v2019.1–0, Bruker AXS Inc., Madison, WI, USA, 2019.
2. Rigaku Oxford Diffraction, CrysAlisPro software system, version 1.171.40.54. Rigaku Corporation, Oxford, 2019.
3. T. D. Kühne, M. Iannuzzi, M. Del Ben, V. V. Rybkin, P. Seewald, F. Stein, T. Laino, R. Z. Khaliullin, O. Schütt, F. Schiffmann, D. Golze, J. Wilhelm, S. Chulkov, M. H. Bani-Hashemian, V. Weber, U. Borštnik, M. Taillefumier, A. S. Jakobovits, A. Lazzaro, H. Pabst, T. Müller, R. Schade, M. Guidon, S. Andermatt, N. Holmberg, G. K. Schenter, A. Hehn, A. Bussy, F. Belleflamme, G. Tabacchi, A. Glöβ, M. Lass, I. Bethune, C. J. Mundy, C. Plessl, M. Watkins, J. VandeVondele, M. Krack and J. Hutter, CP2K: An electronic structure and molecular dynamics software package - Quickstep: Efficient and accurate electronic structure calculations, *J. Chem. Phys.* **2020**, 152, 194103.
4. S. Goedecker, M. Teter and J. Hutter, Separable dual-space Gaussian pseudopotentials, *Phys. Rev. B* **1996**, 54, 1703-1710.
5. P. Hohenberg and W. Kohn, Inhomogeneous Electron Gas, *Phys. Rev.* **1964**, 136, B864-B871.
6. W. Kohn and L. J. Sham, Self-Consistent Equations Including Exchange and Correlation Effects, *Phys. Rev.* **1965**, 140, A1133-A1138.
7. J. P. Perdew, K. Burke and M. Ernzerhof, Generalized Gradient Approximation Made Simple, *Phys. Rev. Lett.* **1996**, 77, 3865-3868.
8. S. Grimme, J. Antony, S. Ehrlich and H. Krieg, A consistent and accurate ab initio parametrization of density functional dispersion correction (DFT-D) for the 94 elements H-Pu, *J. Chem. Phys.* **2010**, 132, 154104.
9. B. K. Maiti, K. Pal and S. Sarkar, Flexible Cu^{I}–Thiolate Clusters with Relevance to Metallothioneins, *Eur. J. Inorg. Chem.* **2007**, 5548-5555.
10. S.-K. Peng, H. Yang, D. Luo, M. Xie, W.-J. Tang, G.-H. Ning and D. Li, Enhancing photoluminescence efficiency of atomically precise copper(I) nanoclusters through a solvent-induced structural transformation, *Inorg. Chem. Front.* **2022**, 9, 5327-5334.
